# Adolescent Alcohol Use in Spain: Connections with Friends, School, and Other Delinquent Behaviors

**DOI:** 10.3389/fpsyg.2016.00269

**Published:** 2016-03-03

**Authors:** Lisa D. Goldberg-Looney, Miriam Sánchez-SanSegundo, Rosario Ferrer-Cascales, Natalia Albaladejo-Blazquez, Paul B. Perrin

**Affiliations:** ^1^Department of Psychology, Virginia Commonwealth University, RichmondVirginia, USA; ^2^Department of Health Psychology, University of AlicanteAlicante, Spain

**Keywords:** adolescent alcohol use, delinquent behaviors, friendship quality, intervention/prevention, peer influences, Spain

## Abstract

This study examined the connections between adolescent alcohol use in Alicante, Spain and variables reflecting adolescents’ academic problems, potentially delinquent behaviors, friends’ alcohol consumption, and friendship quality. Information about alcohol use and a number of school and social variables was collected from adolescent students (*N* = 567) who completed the National Students School-Based Drug Survey in a classroom setting. Results suggested that gender was not significantly associated with alcohol use, although alcohol use increased with age and was more likely for adolescents enrolled in public schools compared to private. After controlling for age and type of school (public vs. private), academic problems explained 5.1% of the variance in adolescents’ alcohol use, potentially delinquent behaviors explained 29.0%, friends’ alcohol use 16.8%, and friendship quality 1.6%. When all unique predictors from these four models were included in a comprehensive model, they explained 32.3% of the variance in adolescents’ alcohol use. In this final model, getting expelled, participating in a fight, going out at night, the hour at which one returns, and the number of friends who have consumed alcohol were uniquely and positively associated with adolescents’ alcohol use. These results provide important information about multi-system influences on adolescent alcohol use in Alicante, Spain and suggest potential areas of focus for intervention research.

## Introduction

Adolescent alcohol consumption has major health and social implications: 12.8% of male and 8.3% of female deaths among 15–29 year olds in Europe result from alcohol-related accidents, health conditions, and risky behaviors (Rehm et al., 2001). Occasional heavy drinking and intoxication common among young adults and adolescents can lead to accidents, injuries, violence, and alcohol poisoning (Ahlström and Österberg, 2004; Llorens et al., 2011). Adolescent alcohol abuse may also be associated with increased predisposition to drink excessively later in life (Jefferis et al., 2005; Windle et al., 2008) when chronic, heavy drinking has been shown to contribute to health conditions, such as alcohol dependence and liver disease (Ahlström and Österberg, 2004; Room et al., 2005). In addition, alcohol use can lead to relationship issues, legal and financial problems, emotional distress, academic problems, sexual assault, and other risk behaviors (Windle, 2000; Ahlström and Österberg, 2004).

Recent studies have found that adolescent drinking is becoming a particularly large health concern in Spain. Traditionally, alcohol has been consumed by Spanish teenagers regularly at family meals, but this behavior is becoming less frequent and instead is being replaced by social drinking with friends on weekends (Villalbí etal., 2006; Delegacion del Gobierno para el Plan Nacional sobre Drogas, 2010). The Spanish Drug Observatory (Ministerio del Interior, 2012) reports that 41.6% of adolescents ages 14–18 years consume alcohol in public parks and squares on the weekends, causing noise disturbances and leaving large amounts of garbage behind. Nearly 20% of these adolescents become involved in disputes while consuming alcohol, 23.3% ride in cars driven by intoxicated drivers, 5.3% operate vehicles under the influence of alcohol, and 7.0% require medical attention for alcohol-related traffic accidents (Ministerio del Interior, 2012).

Past research has identified several school variables that serve as protective or risk factors for adolescent alcohol use (Bryant et al., 2003; Best et al., 2006; Llorens et al., 2011; Vaughan et al., 2011). School bonding, connectedness, and involvement predict lower alcohol use in adolescents. For example, Chapman et al. (2014) examined Australian high school students and found that school connectedness, which they defined as feeling included, supported, and engaged at school, was associated with lower risk taking behaviors, such as alcohol use, as well as students’ willingness to prevent their friends from engaging in risky behaviors. School connectedness has also been associated with students’ relationships and behaviors with friends (Batanova and Loukas, 2012), which may indirectly influence alcohol consumption. Guo et al. (2001) found that strong bonding to school predicted lower alcohol use in an ethnically diverse sample of adolescents in the United States. School involvement has been found to be a protective factor against alcohol use in American college students (Vaughan et al., 2011) but has not been studied in Spanish adolescents. Academic achievement and better grades also correlate with less substance use (Bryant et al., 2003; Zhen-Duan and Taylor, 2014), and school enjoyment is a protective factor against substance use (Bryant et al., 2003; Vaughan et al., 2011).

While school connectedness, involvement, academic achievement and academic enjoyment may protect against alcohol use, truancy (Hibell et al., 2004; Mounteney et al., 2010), suspension (Bryant et al., 2003), lower educational aspirations (Best et al., 2006), poor academic performance (Vaughan et al., 2011), and misbehavior at school (Guo et al., 2001) are associated with increased adolescent alcohol use. Bisset et al. (2007) found that among secondary schools in the UK, those with lower rates of academic success and higher rates of truancy also had higher rates of alcohol use. Another study examining 14–16 year old school children in London found that more than two–thirds of the excessive drinkers had repeated truancy (Best et al., 2006). Others have suggested that truants are a “high-risk population” with a range of social, psychological, cognitive and behavioral problems, including underage drinking (Henry, 2007; Mounteney et al., 2010). Truants have been found to begin drinking at a younger age and have a higher frequency of drinking, levels of binge drinking, and instances of being drunk (Mounteney et al., 2010).

Adolescents who abuse alcohol have also been found to be less likely than those who do not to obtain academic success (Ingles et al., 2013). Failing grades are associated with increased alcohol use in the United States, particularly in Latino/a adolescents (Vaughan et al., 2011). In London, excessive drinking has been linked to lower educational aspirations and worsened performance (Best et al., 2006). A study examining high school students in Granada, Spain found an association between increased alcohol intake and the risk of academic failure (Lopez-Frias et al., 2001).

As with school, the literature supports the role of peers in adolescent alcohol use (Zhen-Duan and Taylor, 2014). Drinking has been conceptualized as a social activity and adolescent drinking is in part related to the closeness of their friendships (Niland et al., 2013). Maladaptive behaviors and substance use by friends predict adolescent substance use (Denault and Poulin, 2012). In fact, one study of Latino/a adolescents demonstrated that 46% of the variance in alcohol use was accounted for by peer use (Segura et al., 2003). Additional studies have suggested that the drinking behavior of friends is the most reliable predictor of adolescent alcohol use (Ahlström and Österberg, 2004). Friends’ perceptions of and behaviors toward alcohol are associated with adolescents’ likelihood of use (Waller et al., 2003), as are adolescents’ perceptions of their peers’ substance use (Bryant and Zimmerman, 2002).

In addition to influences from the school environment and peer group, alcohol consumption has been linked to several other delinquent behaviors (Guo et al., 2001; Best et al., 2006; Llorens et al., 2011). Guo et al. (2001) prospectively followed children beginning at age ten and examined various factors that were associated with increased risk for alcohol abuse at age 21, finding that problem behaviors and antisocial opportunities and involvement were the most consistent predictors. Excessive drinking has been linked to problem behaviors such as impaired social functioning, fighting, and stealing, with those who report drinking excessively also reporting a higher incidence of these behaviors (Best et al., 2006). Other researchers have found associations between alcohol use and misbehavior at school, sexual activity, criminal offenses, and running away from home (Llorens et al., 2011).

The research literature has shown that multiple factors are associated with adolescent drinking behaviors, which have been increasing in Europe generally (Hibell et al., 2009) but in Spain especially (Villalbí etal., 2006; Delegacion del Gobierno para el Plan Nacional sobre Drogas, 2010). However, much of the current literature on adolescent alcohol use comes from the United States or North America, and while some findings may be consistent across countries, countries often have their own cultural norms that impact drinking behaviors (Ahlström and Österberg, 2004). These norms may interact with peer and school variables to influence alcohol use (Batanova and Loukas, 2012). To address the growing issue of adolescent alcohol consumption in Spain, it is important that research be conducted with Spanish adolescents to determine whether similar predictors of alcohol use are operating in Spain. The purpose of the current study was to examine the associations between academic problems, friends’ behaviors, friendship quality, and potentially delinquent social behaviors with alcohol consumption by adolescents in Alicante, Spain. It is hypothesized that academic problems, friends’ drinking behaviors, and potentially delinquent behaviors will be associated with increased alcohol consumption, but that friendship quality will serve as a protective factor and be associated with fewer alcoholic drinks consumed.

## Materials and Methods

### Participants

A total of 640 high school students in Alicante, Spain completed the survey. Inclusion criteria for the students were: (1) being present in the classroom on the day of the survey, (2) the ability to read and complete the questionnaires on their own, and (3) having signed informed consent by their parents allowing participation. Participants were retained in the final sample only if they responded to the primary dependent variable assessing alcohol use and had at least 83% complete data for the independent variables outlined below. Missing data were then imputed using mean substitution, which for low levels of missing data, as in the current study with the 83% cutoff, has been shown to be comparable to more complex methods such as multiple imputation (Parent, 2013). The final sample consisted of 567 students (52.2% female) from public (57%) and private schools in Alicante, Spain ranging in age from 14 to 21 years (*M* = 16.25, *SD* = 0.81).

### Measures

#### ESTUDES

The National Students School-Based Drug Survey (ESTUDES) is a self-administered questionnaire of drug use in adolescents. It contains 90 multiple-choice questions grouped into three modules covering the following domains: Basic Questions (drug use, perceived risk, leisure time, access to and availability of drugs, health-related problems, family drug consumption, knowledge and attitudes regarding drug use); Emerging Drugs (perceived risk of and accessibility to new emerging drugs); and Drug-related Problems (problems experienced due to drug use, severity of dependence, and questions regarding substance-use disorders). The present study focused solely on a selection of the Basic Questions contained in the first module measuring alcohol use among students and friends’ consumption of alcohol.

#### KIDSCREEN-52

The Kidscreeen-52 is a 52-item self-administered questionnaire measuring health-related quality of life in children and adolescents. It yields 10 subscales, but the present study included only the Peers and Social Support subscale, which examines adolescents’ relationships with friends. Items are scored on a 5-point Likert type scale (1 = never, 2 = seldom, 3 = sometimes, 4 = often, 5 = always). Psychometric properties of the Spanish version of the KIDSCREEN-52 have demonstrated adequate validity, reliability, and cross-cultural comparability (Aymerich et al., 2005).

#### Alcohol Consumption Item

The primary dependent variable in this study was participants’ responses to a single item assessing the number of days alcohol was consumed over the past 30 days (“On how many days have you consumed alcohol during the past 30 days?”). Participants selected responses from the following options: 0, 1, 2, 3, 4–5, 6–9, 10–19, 20–29, or 30 days or more. Past research suggests that single item questionnaires for alcohol use and other risky behaviors are often as accurate and sometimes superior to longer, multi-item screening tools (Smith et al., 2009; Barry et al., 2013; Saitz et al., 2014).

### Procedure

The present study was a part of a large-scale study on alcohol abuse funded by the Alicante Municipal Plan on Drug addiction using the National Survey on Drug Use (ESTUDES). As such, the measures were preselected. Students completed the survey as part of an annual nationwide review conducted by the Spanish Drug Observatory for Control and Prevention (DGPNSD), a government organization that provides coordination and technical support for the development of policies and programs on adolescent substance use in Spain. This was performed in a geographic region in the east of Spain with a population of 334,000 people. The study was approved by the University of Alicante (AYTOALICANTE3-13I), and parents provided consent to the participation of their children prior to data collection.

Students 14–21 years old from public and private secondary schools were selected using a stratified random cluster sampling in two stages. In the first stage, all schools were randomly selected with a probability of being selected proportional to the sample size. In the second stage, classes were systematically randomly selected within each school using an enumeration procedure. Students who were present on the day of data collection and who agreed to participate completed the questionnaire anonymously. Data were collected by research assistants during the second and third trimester of the 2013/2014 academic year and lasted from 60 to 90 min.

### Statistical Analyses

A correlation matrix was created to determine which demographic variables were associated with the primary outcome variable, number of days over the past 30 days that alcohol was consumed. Any demographics with significant associations with alcohol consumption were then entered as predictors in Step 1 in each regression in a series of five regressions, which all had the same dependent variable of alcohol consumption. These five regressions included the following categories of predictor variables in Step 2, respectively: (1) academic problems, (2) potentially delinquent behaviors, (3) friends’ alcohol consumption, (4) friendship quality, and (5) all significant predictors from the previous four regressions. The statistical design (hierarchical regression) was chosen in order to understand the associations between adolescent alcohol use and various demographic, social, and academic variables. Using several step-wise models allowed us to control for demographic variables and isolate the unique predictors of the dependent variable.

## Results

### Correlations between Demographics and Alcohol Consumption

Bivariate correlations were calculated to examine the relationships between the demographic variables of age, gender, and type of school (public versus private) with the criterion variable in the successive regression analyses, number of days alcohol was consumed over the past 30 days (**Table 1**). Age and type of school were significantly correlated with alcohol consumption such that alcohol consumption increased with age and was less likely for adolescents enrolled in private schools. Gender was not significantly correlated with alcohol consumption and was excluded from all further analyses. Additional correlation coefficients were calculated indexing the relationships between all other predictor variables in the current study and alcohol consumption (**Table 1**).

**Table 1:** Correlations between alcohol consumption and all predictors.

Predictors	Correlation (*r*) with alcohol consumption
Demographics	
Age	0.25^∗∗^
Public vs. private school	0.10^∗^
Gender	0.03
Academic Problems	
Normally I do not complete any of my homework.	0.04
Have you repeated a course?	0.30^∗∗^
Have you missed class in the past 30 days?	0.13^∗∗^
Have you been expelled in the past 12 months?	0.19^∗∗^
Potentially delinquent behaviors	
Have you run away from home in the past 12 months?	0.14^∗∗^
Have you participated in a fight in the past 12 months?	0.26^∗∗^
Do you go out at night?	0.52^∗∗^
When do you return when you go out at night?	0.41^∗∗^
Friends’ alcohol consumption	
How many of your friends consumed alcohol in the past 30 days?	0.42^∗∗^
How many of your friends got drunk in the past 30 days?	0.37^∗∗^
Friendship quality	
I receive care from my best friend.	0.03
How often do you hang out with your friends?	0.11^∗^
Friends subscale of the KIDSCREEN-52	0.09^∗^

*^∗∗^ Correlation is significant at the 0.01 level (two-tailed). ^∗^ Correlation is significant at the 0.05 level (two-tailed).*

### Academic Problems

The first hierarchical multiple regression tested the hypothesis that academic problems would be positively associated with the number of days in which adolescents consumed alcohol during the past 30 days, after controlling for age and type of school. Age and type of school were entered into the first step of the regression which was statistically significant, *F*(2,549) = 18.91, *p <* 0.001, *R^2^* = 0.064. Age [*β* = 0.25, *p* < 0.001] was a unique predictor of alcohol consumption such that older adolescents more frequently consumed alcohol, but type of school was not (*β* = -0.03, *p* = 0.543). The second step included the following four academic behavior variables: not completing one’s homework, missing class, repeating a course, and being expelled. The addition of this step significantly increased the amount of variance explained in alcohol consumption, *ΔF*(4,545) = 7.82, *p* < 0.001, *ΔR^2^* = 0.051. The overall model with both steps was statistically significant, *F*(6,545) = 11.83, *p* < 0.001, *R^2^* = 0.115. With the addition of the second step, age was no longer a unique predictor (*β* = 0.07, *p* = 0.369). Repeating a course (*β* = 0.19, *p* = 0.007) and getting expelled (*β* = 0.16, *p* < 0.001) were significantly associated with higher alcohol consumption. No other academic problems were significant unique predictors (all *p*s ≥ 0.056).

### Potentially Delinquent Behaviors

A second hierarchical multiple regression was run in the same manner as above but substituting four variables indexing potentially delinquent behaviors in the second step: running away from home, participating in a fight, going out at night, and the hour at which one returns home after going out at night. The addition of this step (after step 1 with age and type of school) significantly increased the amount of variance explained in alcohol consumption, *ΔF*(4,545) = 61.12, *p* < 0.001, *ΔR^2^* = 0.290. The overall model with both steps was statistically significant, *F*(6,545) = 49.81, *p* < 0.001, *R^2^* = 0.347. With the addition of the second step, age was no longer a unique predictor (*β* = 0.07, *p* = 0.068). Participating in a fight (*β* = 0.14, *p* < 0.001), going out at night (*β* = 0.41, *p* < 0.001), and the hour at which one returns (*β* = 0.20, *p* < 0.001) were significantly associated with higher alcohol consumption. Running away from home was not a unique predictor of alcohol consumption (*β* = 0.03, *p* = 0.353).

### Friends’ Alcohol Consumption

A third regression was run in the same manner but substituting in the second step two variables describing friends’ alcohol consumption. The addition of the second step significantly increased the amount of variance explained in alcohol consumption, *ΔF*(2,547) = 59.69, *p* < 0.001, *ΔR^2^* = 0.168. The overall model with both steps was statistically significant, *F*(4,547) = 41.32, *p* < 0.001, *R^2^* = 0.232. With the addition of the second step, age remained a unique predictor (*β* = 0.20, *p* < 0.001). The number of friends who have consumed alcohol in the past 30 days (*β* = 0.31, *p* < 0.001) and the number of friends who have been drunk in the past 30 days (*β* = 0.13, *p* = 0.012) each uniquely predicted alcohol consumption such that greater number of friends engaging in these behaviors were associated with increased participant alcohol consumption.

### Friendship Quality

The fourth hierarchical multiple regression was run in the same manner but substituting three variables related to friendship quality in the second step: receiving care from one’s best friend, spending time with one’s friends, and the Friends subscale from the KIDSCREEN-52. The addition of this step significantly increased the amount of variance explained in alcohol consumption, *ΔF*(3,546) = 3.11, *p* = 0.026, *ΔR^2^* = 0.016. The overall model with both steps was statistically significant, *F*(5,546) = 9.51, *p* < 0.001, *R^2^* = 0.080. With the addition of the second step, age remained a unique predictor (*β* = 0.25, *p* < 0.001). None of the friendship quality variables were unique predictors of alcohol consumption (all *p*s ≥ 0.086).

### Combined

A fifth hierarchical regression included only the significant predictors from the previous regressions, with age in the first step and all other previously significant predictors in the second step. The addition of the second step significantly increased the amount of variance explained in alcohol consumption, *ΔF*(7,543) = 40.92, *p* < 0.001, *ΔR^2^* = 0.323. The overall model with both steps was statistically significant, *F*(8,543) = 42.87, *p* < 0.001, *R^2^* = 0.387. With the addition of the second step, age was no longer a unique predictor (*β* = 0.06, *p* = 0.310). Getting expelled (*β* = 0.08, *p* = 0.032), participating in a fight (*β* = 0.12, *p* = 0.001), going out at night (*β* = 0.33, *p* < 0.001), the hour at which one returns (*β* = 0.14, *p* < 0.001), and the number of friends who consumed alcohol in the past 30 days (*β* = 0.14, *p* = 0.007) were significant predictors such that these indices of academic problems, potentially delinquent social behaviors, and friends’ alcohol consumption were associated with greater alcohol consumption. However, repeating a course and having a greater numbers of friends who got drunk in the past 30 days were not unique predictors of alcohol consumption (*p*s > 0.122).

## Discussion

The current study examined the connections between adolescent alcohol use in Alicante, Spain and variables reflecting adolescents’ academic problems, potentially delinquent behaviors, friends’ alcohol consumption, and friendship quality. After controlling for age and type of school (public vs. private), academic problems explained 5.1% of the variance in adolescents’ alcohol use, potentially delinquent behaviors explained 29.0%, friends’ alcohol use explained 16.8%, and friendship quality 1.6%. When all unique predictors from these four models were included in a comprehensive model, they explained 32.3% of the variance in adolescents’ alcohol use. In this final model, getting expelled, participating in a fight, going out at night, the hour at which one returns, and the number of friends who have consumed alcohol were uniquely and positively associated with adolescents’ alcohol use.

### Demographics and Alcohol Consumption

Past studies have found mixed results in regards to gender and alcohol use. A review of several nations (i.e., the United States, United Kingdom, and Nordic countries) has found little differences between the genders for alcohol use (Ahlström and Österberg, 2004). Similarly, gender was not significantly correlated with alcohol consumption in the present study. Alcohol use did increase with age, consistent with past findings (Llorens et al., 2011) and was less likely for adolescents enrolled in private schools in the bivariate correlation matrix. However, these effects were no longer significant when entered into the final regression model with other previously significant predictors. Past research has found that type of school did not predict alcohol use (Lopez-Frias et al., 2001), corroborating the small and fleeting effect of school that emerged in the bivariate correlation matrix in the current study.

### Academic Problems

It was predicted that academic problems would be positively associated with the number of days on which adolescents consumed alcohol during the past 30 days, after controlling for age and type of school. Getting expelled was significantly associated with higher alcohol consumption, consistent with previous research (Bryant et al., 2003). The literature overwhelmingly suggests that lower educational aspirations (Best et al., 2006), poor academic performance (Vaughan et al., 2011), and academic failure (Lopez-Frias et al., 2001) are associated with increased alcohol use in many nations. In the current study, these constructs were further broken down into the more measurable actions of completing homework and repeating a course. Repeating a course was initially significantly and positively correlated with alcohol use. However, when entered into the final model with all other previously significant predictors, repeating a course no longer uniquely predicted alcohol consumption. Completing one’s homework was not associated with drinking behaviors in the present study.

Despite many previous studies demonstrating an association between truancy and alcohol use (Hibell et al., 2004; Best et al., 2006; Bisset et al., 2007; Mounteney et al., 2010), the current study surprisingly did not find that missing class significantly predicted alcohol consumption. Much of the literature suggesting the link between truancy and alcohol use was conducted with non-Spanish samples, perhaps suggesting that Spanish schoolchildren or school systems differ in some way from those in other countries. It is also interesting to note that the non-significant variables in the current study were behaviors (i.e., not attending class and completing homework), whereas the significant predictors of alcohol use were consequences (i.e., having to repeat a course or being expelled).

### Potentially Delinquent Behaviors

It was hypothesized that potentially delinquent behaviors would be associated with increased alcohol consumption. Participating in a fight, going out at night, and the hour at which one returns were positively associated with alcohol consumption. Previous research also found that alcohol use was related to fighting (Best et al., 2006; Salas-Wright et al., 2014), going out at night, and returning home late at night (Llorens et al., 2011). However, while past studies have found a relatively strong influence of running away from home on alcohol use (Llorens et al., 2011), the current study did not find that running away from home predicted alcohol consumption.

### Friends’ Alcohol Consumption

It was also hypothesized that friends’ alcohol consumption would be associated with increased participant alcohol use. The number of friends who have consumed alcohol in the past 30 days and the number of friends who have been drunk in the past 30 days each uniquely predicted alcohol consumption such that a greater number of friends engaging in these behaviors was associated with increased participant alcohol consumption. This is consistent with past findings showing that peers’ and friends’ alcohol use predicts an individual’s alcohol consumption, as does an individual’s perception of his or her peers’ substance use (Bryant and Zimmerman, 2002; Segura et al., 2003; Waller et al., 2003; Denault and Poulin, 2012). However, when both variables were entered into the final regression examining all previously significant predictors, the number of friends who have been drunk in the past 30 days was no longer significant. This suggests that the amount of alcohol consumed by friends may be less important than the fact that friends are drinking at all.

### Friendship Quality

The final part of the hypothesis was that higher friendship quality would serve as a protective factor against alcohol use. However, none of the friendship quality variables were unique predictors of alcohol consumption. Perhaps this is because strong friendships can have a positive or negative impact on one’s behavior depending on friends’ behaviors (i.e., alcohol use). Strong relationships with friends who are a positive influence (i.e., engage in little to no alcohol use) would likely be associated with decreased alcohol use, while strong relationships with individuals who are a negative influence (i.e., engage in delinquent behaviors including consuming alcohol frequently and/or in large quantities) would probably be associated with increased drinking behaviors. Therefore, a potential reason that no effect was found in the current study may be that the effect of friendship quality on drinking behaviors was washed out by participants having friends who were both a positive or negative influence. This is consistent with previous findings that perceived disapproval of substance use by peers inversely predicted alcohol use, while approval and indifference by peers predicted increased use (Mason et al., 2014).

### Implications

The present study identified academic and peer influences on alcohol use. Additional studies are needed to support the current findings as well as explore causality. If the current findings are supported in future studies with more thorough implementation of measures and perhaps a larger population of adolescents, and a causal link can be established between the variables examined in the study and adolescent alcohol use, school-based interventions may have promise in reducing adolescent alcohol use. Such interventions could draw on social norms and social influence. Many global regions have had success with school-based programs, such as the School Health and Alcohol Harm Reduction Project (SHAHRP) in Australia (McBride et al., 2004), Project ALERT (Ellickson et al., 2003) and ALERT Plus (Longshore et al., 2007) in the United States, and the Unplugged program (Caria et al., 2011) that was tested in seven European countries including Spain via the EU-Dap (European Drug Abuse Prevention) study.

Expanding the focus of school-based programs to include efforts to retain problematic students instead of expelling them and trainings to help students resolve conflicts verbally instead of through physical violence may be ripe targets for intervention research, as both expulsion and participating in a fight were unique predictors of alcohol consumption in the current study. Additionally, based on the current findings, interventions aimed at helping parents of adolescents limit going out at night, establish and enforce an appropriate curfew, and monitor adolescents’ friends’ alcohol consumption may similarly yield promise in helping to curb adolescent drinking in Spain. In fact, Project ALERT, which has been recognized as a model program by the Center for Substance Abuse Prevention, combines instruction, interactive question-and-answer sessions, skill-building activities, group activities, and parental involvement (Ellickson et al., 2003). Cultural tailoring of interventions may be particularly relevant in Spain, where traditional alcohol consumption at family meals is being replaced by social drinking with friends on weekends (Villalbí etal., 2006; Delegacion del Gobierno para el Plan Nacional sobre Drogas, 2010), especially in public parks and squares (Ministerio del Interior, 2001).

### Limitations and Future Directions

There are several limitations of the current study that point to potential areas for future research. One limitation was that only limited demographic information was collected. Future studies may examine additional variables such as race/ethnicity, gender identity, sexual orientation, and socioeconomic status to see how differences in these domains may affect adolescent alcohol use. A second limitation was that many of the variables assessed in the current analysis were derived from single-item measures because the present study was a part of a large-scale study on alcohol abuse funded by the Alicante Municipal Plan on Drug addiction using the National Survey on Drug Use (ESTUDES). While this common technique in epidemiological research, we recognize that it is not as strong as using validated multi-item measures of the constructs. Future studies may instead use validated scales for each of these variables for more comprehensive information. For instance, the dependent variable—adolescent alcohol use—was measured with a single item. A validated scale such as the Alcohol Use Disorders Identification Test (AUDIT; Babor et al., 1992) could be used in future studies. Nonetheless, this concern can be tempered somewhat as researchers have suggested that single item questionnaires for alcohol use and other risky behaviors are often as accurate and sometimes even better than longer, multi-item screening tools (Smith et al., 2009; Barry et al., 2013; Saitz et al., 2014). For instance, Barry et al. (2013) found that a single item asking how often participants typically “got drunk” demonstrated both concurrent and convergent validity with validated measures such as the AUDIT-C and biological measures such as breath alcohol concentration. Saitz et al. (2014) similarly reported that a single question regarding alcohol use was more accurate than the more complex AUDIT-C.

A third limitation was that the data were self-reported and participants could have understated the alcohol use. This concern is mitigated by the fact that the questionnaires were individually and anonymously completed, minimizing the potential for social desirability in responding to questions about alcohol use. Nonetheless, future studies may benefit from incorporating social desirability scales and having collateral reports to verify the self-reported information.

The data were cross-sectional, and as a result, causation cannot be directly inferred. Although the host of variables examined in this study might cause adolescent alcohol use, so too could the reverse causal direction exist. Future research using cross-lagged panel methodologies could more readily tease apart causality. Finally, future research might evaluate the effectiveness and feasibility of culturally tailored interventions for Spanish adolescents and families that target some of the variables found in this study to be association with adolescent alcohol consumption. However, randomized clinical trials investigating whether these interventions actually reduce alcohol consumption need to be conducted before any implications for policy or systematic adoption of programs occur.

## Conclusion

The present study provided important information about multi-system influences on adolescent alcohol use in Alicante, Spain and suggested potential areas of focus for intervention research. Getting expelled from school, participating in a fight, going out at night, the hour at which one returns at night, and the number of friends who consumed alcohol in the past 30 days were uniquely associated with increased adolescent alcohol use. Additional research is needed to support these findings as well as determine a causal direction. If these findings are supported, future research may focus on the development and testing of interventions that target students as well as social and parental influences and may have potential to decrease adolescent alcohol use if they encourage school success, academic achievement, school connectedness, healthy friendships, supervised activities in the home, a curfew, and social opportunities without alcohol. However, such assertions await support from future research.

## Author Contributions

All authors listed, have made substantial, direct and intellectual contribution to the work, and approved it for publication

## Conflict of Interest Statement

The authors declare that the research was conducted in the absence of any commercial or financial relationships that could be construed as a potential conflict of interest.
